# Managing chronic hepatitis C in the difficult-to-treat patient

**DOI:** 10.1111/j.1478-3231.2007.01613.x

**Published:** 2007-12

**Authors:** Nyingi Kemmer, Guy W Neff

**Affiliations:** Division of Digestive Diseases, University of Cincinnati College of MedicineCincinnati, OH, USA

**Keywords:** consensus interferon, hepatitis C, non-responders, pegylated interferon, relapsers, retreatment, ribavirin, sustained virological response

## Abstract

Patients with chronic hepatitis C virus (HCV) infection and disease-related complications – among them cirrhosis and liver failure – pose a particular management challenge. Some of these patients may fail to respond to current therapy (non-responders), and some are affected so severely that treatment puts them at an unacceptable risk for complications. Treatment with pegylated interferon (peg-IFN) plus ribavirin improves hepatic enzyme levels and eradicates the virus in ≈50% of patients; however, a significant number of patients do not respond to therapy or relapse following treatment discontinuation. Several viral, hepatic and patient-related factors influence response to IFN therapy; many of these factors cannot be modified to improve long-term outcomes. Identifying risk factors and measuring viral load early in the treatment can help to predict response to IFN therapy and determine the need to modify or discontinue treatment. Retreatment options for patients who have failed therapy are limited. Retreatment with peg-IFN has been successful in some patients who exhibit an inadequate response to conventional IFN treatment, particularly those who have relapsed. Consensus IFN, another option in treatment-resistant patients, has demonstrated efficacy in the retreatment of non-responders and relapsers. Although the optimal duration of retreatment and the benefits and safety of maintenance therapy have not been determined, an extended duration is likely needed. This article reviews the risk factors for HCV treatment resistance and discusses the assessment and management of difficult-to-treat patients.

As the most common blood-borne pathogen in the United States, hepatitis C virus (HCV) is an escalating healthcare concern (1). Approximately 60–85% of patients acutely infected with HCV progress to chronic disease, defined as the presence of HCV RNA in the blood for more than 6 months (2). The National Center for Health Statistics recently estimated that 1.3% of the US population – or 3.2 million people – have chronic HCV infection (3). The prevalence of infection is highest among patients aged 40–49 years (3); as these individuals age and the disease progresses (Fig. 1) (4), HCV-related complications will become more evident and severe (3, 4).

**Fig. 1:**
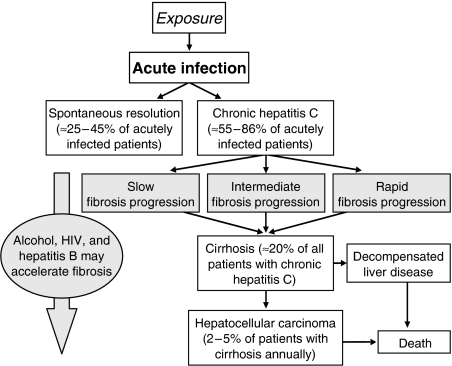
Natural history of hepatitis C virus (HCV) infection. [Reprinted from *Postgraduate Medical Journal*, Lo Ro viii*et al*. ‘Management of Chronic hepatitis C’ 2005; **81**: 378; with permission from BMJ Publishing Group Ltd (4).]

Among individuals with chronic HCV infection, 15–20% progress to end-stage liver disease (5). Cirrhosis and hepatocellular carcinoma, in particular, are life-threatening complications, with an estimated 42% of cirrhosis and 48% of liver cancer cases in the United States, Canada and Cuba attributed to chronic HCV infection (6). HCV-induced liver disease results in 8000–10 000 US deaths each year (7).

Achieving viral eradication is the goal of antiviral therapy and is defined by sustained virological response (SVR; Table 1) (5, 8). The rates of SVR have increased with improvements in antiviral therapy. SVR rates with interferon (IFN) monotherapy are approximately 6–12%, increasing to 38–42% with conventional IFN and ribavirin (RBV), and increasing as high as 55% in major clinical trials of pegylated IFN (peg-IFN) and RBV (9–12). Regardless, the number of patients infected with HCV who are at risk for continued hepatic injury is substantial.

**Table 1:** Treatment outcomes in hepatitis C management (5, 8)

Treatment outcome	Definition
Non-response	<2-log decline in baseline HCV RNA levels after 12 weeks of therapy
Partial response	≥2-log decline in serum HCV RNA occurs, but the virus is detectable after 24 weeks of treatment
Sustained virological response	HCV RNA remains undetectable in the serum 6 months after therapy is discontinued
Relapse	Reappearance of HCV RNA following treatment withdrawal

HCV, hepatitis C virus.

Although some factors that influence SVR rates, such as inadequate dosage, inappropriate management of side effects and early dose reductions, may be corrected, others, such as HCV genotype, baseline viral load, race and age, cannot be specifically modified to improve outcomes (13). Ongoing alcohol and substance abuse has also been shown to contribute to the failure of IFN-based treatment (14–16), as well as the development of hepatocellular carcinoma (17). Identifying the risk factors for poor response can aid clinicians in predicting response and making treatment and retreatment decisions. The patient who does not respond to or who fails prior therapy and is referred for new treatment options poses a particular challenge; for example, a patient who discontinues therapy because of treatment-related adverse effects would have to be carefully evaluated before starting subsequent IFN-based retreatment, especially to determine appropriate management of complications. This article describes viral and host-related risk factors for poor treatment response to initial IFN therapy, evaluates current pharmacological options for the difficult-to-treat HCV-infected population and discusses methods to predict potential treatment failure.

## Viral factors influencing treatment response in hepatitis C virus infection

### Hepatitis C virus population dynamics

Hepatitis C virus exists as a quasispecies, comprising closely related variants that are genetically distinct (18–20). The continuous production of these variants allows HCV to escape host defences and resist clearance by antiviral therapies (18). Thus, patients who have minimal HCV complexity (i.e. small quasispecies sequence) are more likely to achieve SVR than patients with large HCV complexity and significant changes in the quasispecies composition (18, 21). To further understand HCV resistance to antiviral therapies, HCV replicon cell lines were developed with an IFN-resistant phenotype (22). Namba *et al*. (22) suggested that genetic alterations within the cell lines underlie IFN resistance; however, further research is needed to explain fully the mechanisms of antiviral resistance in HCV infection.

### Hepatitis C virus genotype

The HCV genotype, in particular, is a primary predictor of response to IFN therapy. Currently, six major HCV genotypes with multiple subtypes are characterized, and HCV subtypes 1a and 1b are isolated most often in the United States (23). Unfortunately, chronic infection with HCV genotype 1 is associated with greater resistance and lower SVR rates than other genotypes (21, 24–27). In a study of peg-IFN-α-2a and RBV, Fried *et al*. (9) noted that an HCV genotype other than 1 was an independent and significant predictor of SVR (odds ratio, 3.25; 95% confidence interval, 2.09–5.12; *P*<0.001). Nguyen *et al*. (26) also found lower SVR rates with IFN-based therapies in US veterans infected with HCV genotype 1 vs. genotypes 2/3 (13 vs. 61% respectively).

### Viral load

Patients with pretreatment high viral loads have worse long-term outcomes than patients with low loads (24, 28). Evaluating 24-h HCV kinetics, Boulestin *et al*. (24) noted a better response to antiviral therapy when baseline viral loads were <5.55 log_10_ copies/mL. During the first 24 h of IFN-α-2b therapy, reductions in viraemia were greater in patients with low viral loads vs. those with baseline loads ≥5.55 log_10_ copies/mL (1.26±0.14 vs. 0.70±0.22 respectively; *P*=0.016). These investigators also associated large viral loads with slower day 1 viral decay, as reductions ≥0.5 log_10_/24 h were documented in 82% of patients with baseline viral levels <5.55 log_10_ copies/mL (range of viral decay: 0.38–1.72 log_10_ copies/mL) vs. 45% of patients with significant baseline viraemia (range of viral decay: 0.09–2.49 log copies/mL) (24). Similarly, Jessner *et al*. (28) found that responders to peg-IFN-α-2a plus RBV exhibited lower baseline viraemia vs. non-responders (*P*=0.039).

## Host factors influencing treatment response in hepatitis C virus infection

### Fibrosis and cirrhosis

Cirrhosis and cirrhotic liver disease are estimated to develop in 12.5% of patients with a 20-year history of hepatitis C (29). Although patients with advanced fibrosis or early-compensated cirrhosis generally have lower response rates, they can be successfully treated and may achieve SVR (30). In a trial of 1311 patients with advanced liver disease, 63% of all patients and 52% of patients with genotype 1 treated with peg-IFN plus RBV achieved SVRs (31). However, the success of antiviral therapy diminishes in the face of decompensated cirrhotic disease owing to the severity of adverse effects in these severely ill patients (29, 32). In addition, in a subanalysis of the HALT-C (Hepatitis C Antiviral Long-term Treatment Against Cirrhosis) study conducted by Everson *et al*., the presence of advanced fibrosis and cirrhosis was a major independent predictor of non-response to antiviral therapy. This study compared four groups of patients with increasingly severe liver disease, as determined by Ishak scores and platelet counts. SVR rates decreased from 23 to 9% as Ishak scores increased and platelet counts decreased (*P*<0.0001 for trend), confirming the effect of advanced liver disease in antiviral therapy (33).

### Race

Hepatitis C virus kinetics and drug pharmacokinetics are influenced by select demographical and patient-specific characteristics such as race (34). For example, HCV-infected African American patients are less responsive to antiviral therapy than the non-Hispanic white population (35, 36). In 401 patients infected with HCV genotype 1, peg-IFN-α-2a- and RBV-produced SVR rates of 28% for African American patients and 52% for white patients (*P*<0.0001) (37). Breakthrough viraemia (13 vs. 6%; *P*=0.05) was also more common among African Americans. Importantly, rates of serious adverse events, dose reductions and discontinuations were similar between the groups, suggesting another mechanism for the observed efficacy difference.

The mechanism behind a lack of response in African Americans has not been fully elucidated (38). One possible explanation is a high prevalence of HCV genotype 1 among African American patients (88–96%) (36, 39), but this cannot fully explain the insufficient response. Layden-Almer *et al*. (38) noted that compared with white patients, African Americans infected with genotype 1 exhibited significantly lower decreases in first-phase viral RNA (88.6 vs. 98.2% respectively; *P*=0.005), slower elimination of infected cells (0.13/day vs. 0.20/day respectively; *P*=0.006) and smaller declines in mean viral RNA over 1 month (1.15 log_10_ copies/mL vs. 3.61 log copies/mL respectively; *P*=0.001). This suggests that African Americans may have an impaired ability to block viral production.

Fontana *et al*. (40) developed a model to estimate the probability of severe fibrosis in African American and white patients based on commonly available clinical and laboratory parameters. The Ishak fibrosis scores of 205 for white and 194 for African American patients were modelled using simple and multiple logistical regressions. These scores were found to be equally predictive in both groups of patients and may be useful in identifying difficult-to-treat patients in the African American cohort.

### Age

In the elderly population (age >65 years), immunological suppression, chronic disease and concurrent medications adversely affect response and heighten the probability of adverse reactions to antiviral therapy. However, research focusing on the efficacy and safety of antiviral therapy in the older population is limited to a few small, single-centre studies (41–43). In a retrospective cohort study of 84 elderly patients age ≥65 years without genotype 1b or high viral load, 30 patients (35.7%) receiving IFN monotherapy achieved SVRs (41). Eleven patients (13%) withdrew because of adverse events, and univariate analysis showed a higher likelihood of withdrawal owing to adverse events among those age >70 years than those age ≤70 (*P*<0.009). The likelihood of SVR was significantly lower among those with high baseline viraemia (HCV RNA >100 KIU/mL; *P*<0.0001), advanced liver fibrosis (*P*=0.04) and HCV genotype 1 (41). Thus, advanced age alone reduces antiviral effectiveness, but the addition of viral and hepatic risk factors further worsens response.

### Obesity

About 20–37% of HCV-infected patients are obese, a potential barrier to treatment success (44, 45). The body mass index inversely correlates with SVR (46), and serum leptin, which is elevated in obese patients, is a predictor of antiviral treatment resistance in HCV infection with low viraemia (47). One of several explanations developed to explain the interaction between obesity and antiviral therapy response (46) focuses on hepatic steatosis, because obesity is an independent risk factor for fatty liver disease (45, 48, 49). Other explanations include an obesity-triggered inflammatory reaction that decreases response and impairs IFN absorption owing to high levels of subcutaneous fat (44, 46). Regardless of the cause of lower response rates in obese HCV-infected patients (44, 50), weight loss is an important component of treatment, as it may lower elevated liver enzymes and improve liver fibrosis (51). Weight-based dosing of antiviral medications also becomes an important consideration in patients with higher body mass indices (52).

### Hepatitis C virus/human immunodeficiency virus co-infection

An estimated 1 million individuals are human immunodeficiency virus (HIV) positive in the United States alone, and as many as 300 000 are said to be co-infected with HCV (53). Co-infection is associated with substantial morbidity and mortality, including end-stage liver disease, which is the leading cause of death in the hospitalized HIV population (54).

Human immunodeficiency virus co-infection complicates HCV antiviral therapy. Common adverse effects of IFN and RBV therapy, such as depression and anaemia, are often amplified in patients also receiving antiretroviral therapy (55), and the risk of drug–drug interactions is substantial. HCV clearance may be slower in patients co-infected with HIV (56), and relapse is common among co-infected responders (57, 58). In a study by Soriano *et al*. (58) in 89 HIV/HCV-infected patients, 48 (53.9%) exhibited a negative plasma HCV RNA at the end of treatment with peg-IFN-α-2b and RBV, but only 29 (32.6%) achieved SVRs 6 months after treatment discontinuation. In another study (55), 133 patients infected with HCV and HIV were randomly assigned to receive either peg-IFN-α-2a or standard IFN-α-2a with RBV. Although the group receiving the peg-IFN regimen showed higher SVR rates (27 vs. 12%, *P*=0.03), the rate was still lower than the rates reported in patients infected with HCV alone (9, 10, 57).

Management of the co-infected patient must be individualized and should focus on viral suppression with a peg-IFN regimen (59, 60). As stated above, HCV progression is likely enhanced in co-infected patients and, therefore, may justify more aggressive and earlier therapy. Treatment options include HCV-specific regimens, maintenance therapy, alternative IFN formulations and observation (60). Maintenance therapy with low-dose peg-IFN may slow progression of fibrosis, and observation may be an appropriate option for patients with more mild hepatic histology (60). An important issue in this population is whether HCV treatment could enable more extensive use of retroviral agents. This question will likely be answered as more co-infected patients are treated successfully with anti-HCV agents.

### Liver transplantation

Graft re-infection in liver transplantation resulting from chronic HCV infection is common, as the virus is seeded from the bloodstream to the new graft (61). Orthotopic liver transplantation affects response to HCV therapies, with SVR rates of 20–30% (61–68), which are lower than those achieved in non-transplantation patients. A meta-analysis of 48 studies examined the safety and efficacy of both standard IFN and peg-IFN in liver transplant recipients (69). The overall SVR rates were 24% with IFN and RBV and 27% with peg-IFN and RBV; discontinuation rates were 24 and 26%, and pooled rates of graft rejection were 2 and 5% respectively. The slight efficacy advantage for peg-IFN was attenuated by the slight disadvantage in the rates of discontinuation and graft rejections.

Adverse effects also compromise outcomes. Adverse effects can prompt premature treatment discontinuation in up to 50% of patients (61, 70). Severe neutropaenia and the corresponding increased risk of infection and haemolytic anaemia are primary treatment-limiting toxicities associated with IFN plus RBV (61, 71). In 34 HCV-infected transplant recipients treated with peg-IFN-α-2a 180 μg/week for 48 weeks, Chalasani *et al*. (72) documented an SVR rate of only 12% and a withdrawal rate attributed to adverse effects of 30%.

Several investigations regarding optimal regimens for transplant recipients were encouraging (73, 74). One pilot study in 24 post-transplantation patients who did not respond to previous IFN-RBV therapy found that adding amantadine to IFN and RBV led to an SVR rate of 33% (73). In another post-transplantation study, 27 non-responders to recurrent HCV treatment with IFN and RBV were retreated with peg-IFN-α-2b and RBV and compared with 21 untreated patients (74). Only two patients (7%) discontinued therapy because of adverse effects, and eight patients (30%) in the intent-to-treat population achieved an SVR. Interestingly, cyclosporine use (as immunosuppressive therapy) was significantly associated with viral clearance (*P*≤0.03). Fibrosis scores determined on graft biopsy improved in 76% of treated patients and only 5% of untreated patients. Improvement did not correlate with SVR; fibrosis scores improved in 65% of treated patients who did not achieve SVR (74).

### Kidney transplantation

Human immunodeficiency virus infection complicates the treatment of kidney transplantation candidates and graft recipients. Up to 32.1% of patients on maintenance dialysis are anti-HCV positive (75–78), as are 6.8% of adult cadaveric renal graft recipients (79). HCV infection is an independent risk factor that increases the risk of death among dialysis patients up to 2.39-fold (80–82) and increases mortality rates among transplant recipients (80, 81). Chronic hepatitis C is also associated with mixed essential cryoglobulinaemia (83), increasing post-transplantation morbidity by enhancing the risk of *de novo* or recurrent HCV-associated glomerulopathies (84–88). Recurrence of HCV-associated kidney disease can adversely affect graft survival and has been linked to higher serum creatinine levels (85, 89).

Data support treating patients who have chronic hepatitis C and are awaiting kidney transplantation (90, 91), improving both renal histology and biochemical markers of renal function (90). IFN monotherapy is the treatment of choice in HCV-positive dialysis patients awaiting transplantation (92, 93); data are limited on peg-IFN in dialysis patients (94–97). In addition, RBV is generally avoided in dialysis patients, because it may induce haemolytic anaemia (98). Two meta-analyses found that IFN monotherapy produced SVR rates of 33–39% (92, 99). RBV may also be indicated in patients with HCV-related glomerulopathy because it may reduce rates of proteinuria. Importantly, virological relapse rates are very low in dialysis patients who achieve an SVR before transplantation (91). Pre-transplantation treatment may also prevent postoperative complications such as fibrosing cholestatic hepatitis (91).

Amantadine monotherapy was not efficacious in treating HCV infection in renal transplantation patients, showing no effect on HCV viraemia or liver histology (100). The addition of amantadine to RBV was also not superior to RBV in renal transplantation patients with chronic hepatitis C, perhaps because of the poor tolerability of both medications in patients with impaired renal function (101).

Routine antiviral therapy for patients after kidney transplantation is not recommended because of risk of graft rejection (102–106). Exceptions may include patients with HCV-associated glomerulonephritides to prevent graft loss. Patients with advanced fibrosis may also receive treatment to prevent death from liver-related complications.

## Other factors

Patient non-compliance and incorrect medication administration may be modifiable risk factors for treatment failure. Illicit drug or alcohol abuse are associated with non-compliance, and adverse events prompt treatment discontinuation in up to 14% of patients receiving peg-IFN regimens (2). The common, occasional and rare adverse events seen with IFN and RBV are listed in Table 2 (107). Similarly, psychiatric disorders (e.g. depression) present before treatment initiation or resulting from treatment may compromise compliance and cause early treatment discontinuation (8, 26).

**Table 2:** Adverse effects of interferon and ribavirin

Interferon
Common (≥10%)
Mild bone marrow suppression (anaemia, leucopaenia, thrombocytopaenia)
Depression
Insomnia
Fatigue and irritability
Weight loss/anorexia
Fever, myalgia, headaches, and flu-like symptoms
Injection-site irritation
Nausea/vomiting and diarrhoea
Occasional (2–9%)
Retinopathy (usually not clinically significant)
Exacerbation of autoimmune condition (e.g. hepatitis, thyroiditis, rheumatoid arthritis, psoriasis)
Congestive heart failure and arrhythmias
Rare (≤1%)
Severe bone marrow depression
Seizures
Tinnitus and hearing loss
Hyperglycaemia
Renal failure
Pneumonitis
Ribavirin
Common (≥10%)
Haemolytic anaemia (dose dependent)
Fatigue
Rash and pruritis
Nasal stuffiness
Cough

‘Hepatitis C: a review for primary care physicians’. Adapted from *CMAJ*; **174**: 649–659 by permission from the publisher. © Canadian Medical Association (107).

## Treatment of difficult-to-treat human immunodeficiency virus patients

Preventing complications is a prominent consideration in the management of HCV infection, and aggressive attempts are required to treat patients at risk for complications. The natural history of the disease should also be a major factor in deciding on a course of therapy, as patients with mild or no fibrosis may not require such aggressive therapy.

Patients exhibiting any of the multiple risk factors, influencing response to antiviral therapy, pose a challenge in the management of chronic hepatitis C. Although a regimen of peg-IFN and RBV is the standard of care, consensus IFN (CIFN) and RBV also may also be efficacious in treatment-naïve patients at risk for poor response. In an open-label, prospective study by Sjogren *et al*. (108), 128 treatment-naïve patients with chronic HCV infection were randomized to receive CIFN 15 μg or IFN-α-2b 3 MU three times weekly plus RBV 1000 mg/day for 48 weeks. The results showed a substantial difference in SVR rates: At 72 weeks, 57% of the CIFN treatment group was HCV RNA negative compared with 40% of the IFN-α-2b group (*P*=0.052). Subgroup analyses showed significantly higher SVR rates with CIFN in patients with high baseline HCV RNA (≥800 000 IU/mL) or HCV genotype 1 or both (Table 3). The investigators concluded that CIFN should be considered for treatment-naïve patients, particularly those with high viral loads or genotype 1 infection (108).

**Table 3:** Sustained response to consensus interferon/ribavirin vs. interferon-α-2b/ribavirin by hepatitis C virus genotype, ethnicity, gender and body weight

	Consensus interferon/ribavirin (*n*=63)	interferon-α-2b/ribavirin (*n*=65)	*P*-value
All genotypes			
<800 000 IU/mL	12/21 (57%)	14/26 (54%)	0.82
≥800 000 IU/mL	24/42 (57%)	12/39 (31%)	0.017*
Genotype 1			
<800 000 IU/mL	5/13 (38%)	9/20 (45%)	0.70
≥800 000 IU/mL	13/28 (46%)	4/28 (14%)	0.0089*
Genotype-non 1			
<800 000 IU/mL	7/8 (88%)	5/6 (83%)	0.82
≥800 000 IU/mL	11/14 (79%)	8/11 (73%)	0.70
Race			
White	28/42 (67%)	16/40 (40%)	0.015*
Non-white	8/21 (38%)	10/25 (40%)	0.89
Sex			
Men	19/43 (44%)	17/44 (39%)	0.59
Women	17/20 (85%)	9/21 (43%)	0.005*
Weight			
<75 kg	15/19 (79%)	10/20 (50%)	0.059
≥75 kg	21/44 (48%)	16/45 (36%)	0.24

[Adapted with permission of Springer Heidelberg from *Digestive Diseases & Sciences;***50**: 227–232. © 2005 (108).]

* Statistically significant.

The efficacy of high-dose peg-IFN was evaluated in the RENEW trial (RE-treatment of Non-responders with Escalating Weight-based Therapy trial), in which 704 non-responders to IFN plus RBV therapy were randomized to receive peg-IFN-α-2b 3.0 or 1.5 μg/kg/week plus RBV 12–15 mg/kg/day (109). SVRs were achieved in 17% of the 3.0 μg/kg group vs. 12% of the 1.5 μg/kg group (*P*=0.03; intent-to-treat analysis). Safety and tolerability were similar (115).

**Fig. 2:**
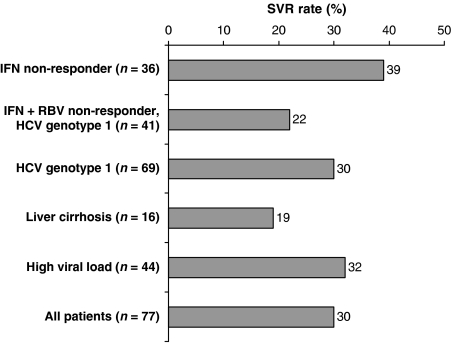
Sustained virological response per baseline viral and patient characteristics. [Reprinted from *Journal of Hepatology*, Cornberg M *et al.*‘Treatment with daily consensus interferon (CIFN) plus ribavirin in non-responder patients with chronic hepatitis C: a randomized open-label pilot study.’ 2006; **44**: 296. With permission from the European Association for the Study of the Liver (110).]

Consensus IFN, another option for the retreatment of chronic HCV infection, is associated with SVR rates of 26–30% in non-responders and rates as high as 58% in relapsers (110–112). Cornberg *et al*. (110) conducted an open-label pilot study of CIFN and RBV in 77 patients who did not respond to standard IFN regimens (90% of patients had HCV genotype 1). CIFN was given in an induction dose of 18 μg/day for 8 weeks, followed by 9 μg/day for 40 weeks or as a standard dose of 9 μg/day for the full 48 weeks. RBV dose was weight based: 1000 mg/day for <75 kg or 1200 mg/day for >75 kg. The SVR rate was 30% (23/77) of the entire population and 22% (9/41) of prior non-responders with HCV genotype 1. Surprisingly, the SVR rate was 28% with the 18/9 μg/day induction regimen but 32% with the 9 μg/day regimen. In subset analyses, investigators noted the greatest response among patients previously treated with IFN monotherapy and the poorest response among those with liver cirrhosis (Fig. 2) (110).

The DIRECT trial (Daily-Dose Consensus Interferon and Ribavirin: Efficacy of Combined Therapy trial) was a phase 3, open-label, multicentre study investigating daily CIFN in 343 previous non-responders to peg-IFN and RBV (113). Patients were randomized to receive either CIFN 9 μg/day and RBV (1000–1200 mg/day) or CIFN 15 μg/day and RBV. The majority of patients had evidence of bridging fibrosis or cirrhosis on biopsy, and the mean washout period was 485–535 days. Viral response rates at week 48 were 16% [transcription-mediated amplification (TMA) assay] and 22% [branched DNA (bDNA) assay] for patients receiving CIFN 9 μg, and 19% (TMA) and 25% (bDNA) for those receiving CIFN 15 μg. Viral response was lower in patients with higher fibrosis scores. Among patients receiving CIFN 9 μg, end-of-treatment responses were noted in 19% with fibrosis scores of F0–F2 (TMA), 16% with F3 and 8% with F4. Among patients receiving CIFN 15 μg/day, end-of-treatment responses were noted in 28, 19 and 6% of patients respectively. The end-of-treatment response rate was lower for patients who underwent longer washout periods. The effect of fibrosis score and washout period in this study may require further investigation.

Treatment duration is critical in complicated chronic hepatitis C infection. A peg-IFN plus RBV regimen should be continued for 24 weeks in treatment-naïve patients infected with HCV genotypes 2 and 3 and for at least 48 weeks in those infected with HCV genotype 1 (2). Retreatment of poor responders requires longer durations of therapy, although the optimal duration is not yet determined. Study durations for peg-IFN or CIFN in non-responders and relapsers have ranged from 24 to 72 weeks (108, 110, 112, 114–117).

Long-term maintenance therapy with IFN may prevent relapse in treatment-resistant patients. Small studies showed that this long-term treatment strategy can maintain biochemical and virological responses and prevent liver histological progression in patients with an initial partial response to IFN therapy (118, 119). In one maintenance study, 53 patients who had normalized ALT values but positive HCV RNA titres after 6 months of IFN-α-2b 5 MU three times weekly were randomly assigned to continue a reduced dose of IFN 3 MU three times weekly or stop therapy (119). During the initial antiviral treatment, significant reductions in serum ALT levels, viral load and hepatic inflammation were noted (*P*<0.05 vs. baseline for all measures). These improvements were sustained in patients receiving maintenance therapy. In contrast, serum ALT, HCV RNA titres and hepatic inflammation returned to baseline values after IFN withdrawal, and significant increases in the mean hepatic inflammatory scores (*P*=0.004 vs. maintenance group) and worsening hepatic histology per Knodell scoring were seen in patients not receiving long-term therapy (*P*<0.01 vs. maintenance group) (119).

Several trials are assessing the efficacy of peg-IFN alone or with RBV as maintenance therapy for chronic hepatitis C (120). The ongoing HALT-C trial is designed to determine the efficacy and safety of low-dose peg-IFN-α-2a 90 μg/week for 3.5 years in chronic hepatitis C patients with bridging fibrosis or cirrhosis and persistent viraemia despite previous IFN therapy (121). The results of such trials should clarify the role of long-term IFN therapy in difficult-to-treat patients with chronic hepatitis C.

Drugs in development include small molecules such as the protease inhibitor, polymerase inhibitor and toll-like receptor drug classes. While many of these drugs seem to hold promise as either a primary or an adjunctive treatment for patients with chronic hepatitis C, they are years from market and their safety and efficacy are uncertain in difficult-to-treat patients (122). In the meantime, the IFNs will continue to form the backbone of HCV therapy for both initial therapy, retreatment and maintenance therapy.

### Predicting treatment response in hepatitis C virus

Achieving viral negativity by week 12 of therapy is highly predictive of SVR (9). In a retrospective analysis, the SVR rate at 72 weeks was 67% among adults with chronic hepatitis C who achieved early virological response with peg-IFN-α-2a and RBV at week 12 (HCV RNA negative or 2 log decrease) (123). In another study, the SVR rate was only 3% among those who did not show a 2-log decline or achieve undetectable HCV RNA at week 12 (9). Therefore, adequate monitoring of patients is important throughout therapy as it allows identification of patients with inadequate treatment response who may benefit from early introduction of alternative therapies.

Methods that evaluate potential treatment outcomes could help determine the most appropriate course of HCV therapy. Hayashida *et al*. (124) developed a pretreatment predictive algorithm based on liver messenger RNA expression profiles rather than viral factors. In this validation study, the algorithm accurately predicted sustained/transient response and non-response rates of 97 and 86% (*P*<0.00001), respectively, with IFN monotherapy and 97% (*P*<0.0001) and 87% (*P*<0.05), respectively, with combination antiviral therapy.

While this algorithm may be useful in initially tailoring antiviral therapy, its utility is limited to the academic setting. In contrast, modelling HCV kinetics within the first few weeks of IFN therapy is used in the ambulatory care setting and is key to maximizing individual patient outcomes (125). Terrault *et al*. (126) reported that the probability of a non-sustained response increases as the values for viral load and log_10_ decline in viral load move further away from designated thresholds. Similar findings have been documented by other studies, and hence a <2-log_10_ decline in HCV RNA at 12 weeks is highly predictive of a poor response to any combinations of antiviral therapy (9, 25). Given available data and the importance of predicting response to antiviral treatment, the National Institutes of Health, in the 2002 Consensus Statement on Hepatitis C Management, recommended that only patients exhibiting at least a 2-log_10_ decline in HCV RNA after 12 weeks of combination therapy should continue long-term treatment (2).

Although the 12-week stopping rule is used to guide therapy, measures of absolute viral load and log decline in viral load earlier in the course of HCV therapy may more accurately predict an SVR as well as a non-response. In a study of 351 HCV-infected patients receiving standard treatment with IFN plus RBV, a viral load >100 000 IU/mL after 4 weeks of treatment and a viral load >10 000 IU/mL or a <2 log_10_ decline after 8 and 12 weeks had a negative predictive value >95% for a non-SVR (Table 4) (126). At these thresholds, the negative predictive value remained >95%, regardless of HCV genotype. Therefore, stopping therapy at 4 weeks after a negative viral response may help to avoid treatment-related adverse events and allow earlier re-evaluation of retreatment options.

**Table 4:** Positive and negative values, clinical sensitivity and specificity of viral load, and log-decline predictors of sustained and non-sustained virological response*

		Negative predictive value†		Positive predictive value‡		Clinical specificity§	Clinical sensitivity¶
Week	Prediction rules	% (95% CI)	*n*	% (95% CI)	*n*	% (95% CI)	% (95% CI)
4	100 000 IU/mL	96.6 (88.3–99.6)	57/59	55.5 (49.2–61.7)	142/256	33.3 (26.3–40.9)	98.6 (95.1–99.8)
	1-log decline∥ or <1000 IU/mL	94.0 (86.7–98.0)	79/84	60.2 (53.5–66.5)	139/231	46.2 (38.6–54.0)	96.5 (92.1–98.9)
8	10 000 IU/mL	98.7 (92.8–100.0)	74/75	66.5 (59.6–73.0)	135/203	52.1 (43.6–60.6)	99.3 (96.0–100)
	2-log decline or <1000 IU/mL	97.5 (91.2–99.7)	77/79	67.3 (60.3–73.8)	134/199	54.2 (45.7–62.6)	98.5 (94.8–99.8)
12	10 000 IU/mL	97.1 (90.1–99.7)	68/70	59.1 (52.6–65.3)	143/242	40.7 (33.2–48.6)	98.6 (95.1–99.8)
	2-log decline or <1000 IU/mL	97.4 (90.9–99.7)	75/77	60.9 (54.3–67.1)	143/235	44.9 (37.2–52.8)	98.6 (95.1–99.8)

[Adapted with permission of Blackwell Publishing Ltd from *Journal of Viral Hepatitis*. 2005; **12**: 465–472. © 2005.]

* All patients included.

† Percentage of patients with actual non-sustained virological response of those who were predicted to have non-sustained virological response (i.e. HCV RNA positive at 6 months post-therapy).

‡ Percentage of patients with actual sustained virological response of those who were predicted to have sustained virological response (i.e., HCV RNA negative at 6 months post-therapy).

§ Percentage of patients correctly predicted by the test as having non-sustained virological response.

¶ Percentage of patients correctly predicted to have a sustained virological response of all patients having sustained virological response.

∥ None of the log decline rules at week 4 attained a negative predictive value of >95%. The 1-log decline had the highest negative predictive value.

CI, confidence interval.

Viral kinetics can accurately predict treatment response; however, questions about when to assess response remain. Intuitively, treatment earlier rather than later is desirable. Prediction of non-response is possible after a single dose of IFN in patients infected with HCV (28, 127). Carlsson *et al*. (127) noted that patients achieving SVR with standard IFN 3 MU plus RBV had a 79% decline in HCV RNA levels following the first IFN dose. Similarly, in a study by Jessner *et al*. (28) in 22 patients with chronic HCV genotype 1, a change in viral load of >1.4 log_10_ 24 h after treatment with IFN-α-2a 9 MU had a 100% specificity in predicting an SVR after 1 year of combination antiviral treatment and a 100% sensitivity and 81% specificity for a non-response prediction.

In contrast, 24-h measures of viral load after administration of peg-IFN-α-2a are not predictive of treatment outcomes. Pegylation of IFN changes the drug's pharmacokinetics, and maximum plasma concentrations persist over a longer period, half-life is extended and clearance is reduced (128, 129). Thus, 24 h is insufficient when evaluating treatment response with peg-IFN; however, a period of 2 weeks is an effective time to assess antiviral responsiveness in treatment-naïve patients receiving peg-IFN regimens (130). Ouzan *et al*. (130) administered peg-IFN-α-2a 180 μg/week plus RBV 1 g/week to 20 treatment-naïve patients infected with HCV genotype 1. They noted that a viral load decline >1.39 log_10_ copies/mL at week 2 was associated with a positive predictive value of 91% and a negative predictive value of 89%. After 4 weeks of treatment, the negative predictive value increased to 100% with a viral drop threshold of 2.81 log_10_ copies/mL. Findings from a study by Carlsson *et al*. (25) suggest that treatment outcomes can be predicted even earlier in the treatment course. After 1 week of peg-IFN-α-2a 180 μg/week in patients with HCV genotype non-1, a 2-log_10_ copies/mL decline in HCV RNA levels was associated with an 89% positive predictive value for SVR. In this investigation, the negative predictive value of a 2-log_10_ decline in viral load was only 43% at week 1 but increased to nearly 100% after 12 weeks of treatment (25).

**Fig. 3:**
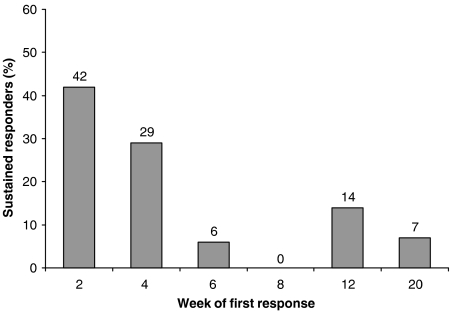
Percentage of sustained responders who showed first viral response by week. [Reprinted with permission of John Wiley & Sons Inc., *Hepatology*, 1998; **28**, 1411–1415. © 1998 American Association for the study of Liver Diseases.]

A retrospective analysis evaluated whether early declines in HCV RNA would predict treatment efficacy of CIFN (131). In the analysis, two trials of CIFN 9 or 15 μg in treatment-naïve patients or non-responders/relapsers were included for evaluation. Early declines in viral RNA were associated with SVR in treatment-naïve patients (Fig. 3) (131). Measures of viral load were unavailable until week 8 for the retreatment group; however, patients who experienced SVR responded early in the treatment course, as 88% of responders had undetectable HCV RNA levels by week 8 and 95% had cleared the virus by week 16. In this analysis, 80% of sustained responders, including treatment-naïve and retreatment patients, had undetectable levels of virus by week 8. This percentage increased to 95% by week 12 in treatment-naïve patients and by week 16 in retreatment patients (131). Based on available data, patients with early HCV RNA clearance are more likely to experience SVR than patients exhibiting later declines in viral load. Thus, early assessment of viral kinetics may help to predict sustained response to IFN therapy and determine the value of continuing therapy sooner, rather than later, in the treatment course.

## Conclusions

Multiple factors related to HCV may negatively affect treatment outcomes and complicate management of patients with hepatitis C. Unfortunately, because most of these factors cannot be modified, a significant number of patients will not respond to antiviral therapy or will relapse following treatment withdrawal. Recognizing viral, hepatic and demographical factors that foster treatment resistance in HCV infection can alert clinicians to the potential for poor long-term outcomes with IFN plus RBV treatment. Similarly, methods (e.g. measuring viral load early in the treatment course) may help to predict poor response and determine the need for treatment modifications.

Therapeutic options for treatment-resistant patients are limited, but retreatment with peg-IFN plus RBV or CIFN can produce SVR in complicated HCV infections. Additionally, preliminary findings suggest limited benefits of maintenance IFN therapy in patients remaining HCV RNA positive with antiviral therapy. Although potentially beneficial, the cost, safety and risk of non-compliance could limit this approach.
